# First year physical activity findings from turn up the HEAT (Healthy Eating and Activity Time) in summer day camps

**DOI:** 10.1371/journal.pone.0173791

**Published:** 2017-03-28

**Authors:** R. Glenn Weaver, Keith Brazendale, Jessica L. Chandler, Gabrielle M. Turner-McGrievy, Justin B. Moore, Jennifer L. Huberty, Dianne S. Ward, Michael W. Beets

**Affiliations:** 1 University of South Carolina, Department of Exercise Science, Columbia, South Carolina, United States of America; 2 University of South Carolina, Department of Health Promotion, Education, and Behavior, Columbia, South Carolina, United States of America; 3 Wake Forest School of Medicine, Department of Family and Community Medicine, Winston-Salem, North Carolina, United States of America; 4 Arizona State University, School of Nutrition and Health Promotion, Phoenix, Arizona, United States of America; 5 University of North Carolina at Chapel Hill, Department of Nutrition, Chapel Hill, North Carolina, United States of America; TNO, NETHERLANDS

## Abstract

**Background:**

Summer day camps (SDCs) serve 14 million children yearly in the U.S. and aim to provide participating children with 60 minutes of moderate-to-vigorous physical activity (MVPA). This study evaluated an intervention designed to increase the percent of children meeting this MVPA guideline.

**Design:**

Two-group, pre-post quasi-experimental.

**Setting/Participants:**

Twenty SDCs serving 1,830 children aged 5–12 years were assigned to MVPA intervention (n = 10) or healthy eating attention control (n = 10).

**Intervention:**

The STEPs (Strategies to Enhance Practice) intervention is a capacity-building approach grounded in the Theory of Expanded, Extended and Enhanced Opportunities. Camp leaders and staff receive training to expand (e.g., introduction of activity breaks/active field trips), extend (e.g., schedule minimum of 3 hours/day for PA opportunities), and enhance (e.g., maximize MVPA children accumulate during schedule activity) activity opportunities. Camps in the comparison condition received support for improving the types of foods/beverages served.

**Main outcome measures:**

Percent of children accumulating the 60min/d MVPA guideline at baseline (summer 2015) and post-test (summer 2016) measured via wrist-accelerometry.

**Results:**

Multilevel logistic regression conducted fall 2016 indicated boys and girls attending intervention SDCs were 2.04 (95CI = 1.10,3.78) and 3.84 (95CI = 2.02,7.33) times more likely to meet the 60min/d guideline compared to boys and girls attending control SDCs, respectively. This corresponded to increases of +10.6% (78–89%) and +12.6% (69–82%) in the percentage of boys and girls meeting the guideline in intervention SDCs, respectively. Boys in comparison SDCs increased by +1.6% (81–83%) and girls decreased by -5.5% (76–71%). Process data indicated intervention SDCs successfully extended and enhanced PA opportunities, but were unable to expand PA opportunities, compared to control SDCs.

**Conclusions:**

Although substantial proportions of children met the MVPA guideline at baseline, no SDCs ensured all children met the guideline. This intervention demonstrated that, with support, SDCs can help all children in attendance to accumulate their daily recommended 60min MVPA.

**Trial registration:**

ClinicalTrials.gov NCT02161809

## Introduction

The largest providers of summer day camps (SDCs) in the U.S., such as the YMCA of the USA [1], the Boys and Girls Clubs of America [2], and the National Recreation and Parks Association [3], recently adopted physical activity guidelines that call for SDCs to ensure all children accumulate 60 minutes/day of moderate-to-vigorous physical activity (MVPA) while in attendance. This guideline has the potential to impact the health of the 14 million youth that attend SDCs annually in the U.S. [4] Moreover, this guideline is especially important as summer (commonly 2–3 months) has been identified as a “window of vulnerability” for children in terms of health outcomes. Recent studies have clearly demonstrated that children gain excess weight [5, 6] and lose fitness [7, 8] over the summer when compared to the school year. Thus, interventions targeting settings during times when children are vulnerable to unhealthy behaviors are critically important.

Few studies have examined children’s accumulation of MVPA during SDCs, and a dearth of studies evaluating interventions to increase children’s physical activity in this setting exist. Those studies that do exist [9–11] report widely different levels of MVPA ranging from 36.9 min/d [9] to 86.0 min/d [11]. These varying estimates are due to the use of different measurement devices (e.g., pedometers vs. accelerometers), and small sample sizes (e.g., 184 children in 5 SDCs) [11]. Thus it is challenging to estimate the amount of MVPA camps currently provide the children that attend. Further, studies that have targeted increasing children’s physical activity during SDCs have focused almost exclusively on creating new, specialty camps that target relatively small, mostly middle to upper income, segments of the population. For instance, several studies have created residential weight loss camps to evaluate their efficacy in reducing weight in overweight youth [12–14] or evaluated the effectiveness of a SDC that was created for a small number of low income minority girls (N = 19) [10]. Only one study to date has evaluated the effectiveness of an intervention designed to enhance the amount of MVPA accumulated by children attending pre-existing non-specialty SDCs [15, 16]. While the study did not have a comparison group and was limited to four SDCs, it showed promising increases in staff promotion of physical activity during the SDC and corresponding increases in the percent of children engaged in MVPA during physical activity opportunities [15, 16]. However, because of the use of systematic observation, estimation of the proportion of children meeting the 60 min/d of MVPA guideline is not possible.

Given the potential impact of SDCs on the MVPA of attending youth and potential for excessive weight gain and loss of fitness during the summer, especially in the most vulnerable youth, it is critically important to understand the dose of MVPA these SDCs are providing for children who attend. This information can inform the development of interventions to maximize SDCs impact on the achievement of the 60 min/d MVPA guideline. The objective of this study was to evaluate the implementation and effectiveness of a theory-based, multi-component adaptive intervention in SDCs. The goal of the intervention was to increase the percentage of children meeting the 60 min/d MVPA guideline while in attendance. This study is reported in accordance with the Transparent Reporting of Evaluations with Nonrandomized Designs (TREND) statement [17].

## Methods

### Study population, setting, and design

A total of 20 SDCs operated by nine organizations were recruited to participate in this study. SDCs were identified from a list of 62 unique SDCs operated by 16 organizations in one southeastern U.S. state. The list of SDCs was compiled via internet searches for SDCs and summer programs operating within a 120-minute drive of the principal investigator’s (PIs) university. SDCs were defined as operating Monday-Friday, for at least 8 hours per day (8AM to 6PM), for a minimum of 8 weeks during the summer. Further, SDCs could not provide accommodations for children to stay overnight and could not have a singular focus such as sports, arts, or academics. SDCs serving fewer than 40 children were also excluded from participation. Of the 62 SDCs identified 44 met inclusion criteria. Of the 18 that did not meet inclusion criteria, 11 did not serve at least 50 children, 5 were specialty SDCs, and 2 were overnight camps. Organizations were randomly selected from this list and invited to participate in the study. A total of nine organizations operating 26 unique programs agreed to participate. All children, staff, and SDC leaders at the participating SDCs were eligible to participate. However, children that were unable to engage in physical activity without an assistive device (e.g., wheelchair, crutches) were excluded from participation in the MVPA outcome assessment using accelerometry. See **Table 1**for complete details on the characteristics of the SDCs included in this study. All study procedures were approved by the University of South Carolina institutional review board.

**Table 1:** Characteristics of participating summer day camps (SDCs) and children by intervention and control at baseline.

	Baseline (Summer 2015)					Outcome (Summer 2016)				
	Intervention		Control		P difference ^b^	Intervention		Control		P difference ^b^
SDC Characteristics										
Total children (*n*)	904		926			797		762		
Average enrollment (n)	90.4	±46.4	92.6	±47.9	0.91	79.7	±32.2	76.2	±36.2	0.82
Girls (%)	46.3		43.6		0.27	52.2		37.9		**0.002**
Physical Activity Space (ft^2^)										
Indoor	10,390.4	±5,135.7	13,058.5	±4,928.1	0.13	-		-		-
Outdoor	172,226.0	±180,402.9	259,411.0	±170,088.8	0.28	-		-		-
Population in Poverty, Census 2014 (%)	15.1	±8.9	11.8	±4.7	0.32	-		-		-
Location (*n*)					**0.03**					
School	2		3			-		-		-
Faith/church	3		0			-		-		-
Community (e.g., recreation center)	2		7			-		-		-
Other (e.g., strip mall, military base)	3		0			-		-		-
Temperature (°F)										
High	95.2	±2.9	95.8	±3.3	0.39	95.5	±3.4	93.6	±4.5	**0.03**
Low	74.7	±2.9	74.7	±2.7	0.96	76.8	±3.1	76.8	±3.7	0.95
Child Characteristics										
Race/Ethnicity (%)					0.15					0.06
White non-Hispanic	38.2		13.2			37.5		17.7		
African American	54.4		80.8			56.4		78.9		
Other	7.5		6.0			6.0		3.5		
Age (years, M, SD)	7.9	±2.0	8.0	±1.8	0.32	8.0	±1.8	7.8	±2.0	0.55
Children Assessed via Accelerometry (*n*)										
Children wearing accelerometer	582		536			614		536		
Average number of days	2.2		2.3			2.2		2.4		
Children meeting accelerometer inclusion criteria ^a^	576		512			602		519		
Average hours of wear time	8.5	±0.3	8.6	±0.5	0.20	8.5	±0.3	8.3	±0.4	0.29

^a^ Accelerometer inclusion criteria wear time ≥240 minutes per day.

^b^ P-value for comparison between intervention and control at baseline and outcome. Continuous variables at the SDC-level compared using two-sample t-tests or Mann Whitney U-test where appropriate; categorical variables compared using χ2 tests.

Statistically significant differences are bolded.

The information presented herein represents the first year physical activity outcomes from baseline (summer 2015) to end of first year (summer 2016) of a three-year intervention. The study used a repeated cross-sectional, quasi-experimental design. This design is consistent with recent large scale interventions that target changes at a program level [18, 19]. Following baseline data collection, the 20 SDCs were assigned to one of two conditions 1) physical activity intervention 2) or healthy eating intervention (attention control).

### Assignment to study arm

Initially the study was designed as a matched pair group RCT. The intention was to match SDCs using baseline activity levels, foods served, and child demographics attending the SDCs and then randomly assign SDCs to either the physical activity intervention or healthy eating attention control. However, it was not possible to randomly assign SDCs that would result in comparable groups due to the overlap in the third party providers of meals contracted by the SDC organizations (a focus of the healthy eating intervention in the comparison group). Thus, organizations and their SDCs were equally divided based upon the separation of food providers into two groups of 10 SDCs each and assigned to receive either the physical activity or healthy eating intervention. Participating SDCs were not blinded to which study arm they had been assigned.

### Intervention

The intervention to meet the guideline of 60 min/d of MVPA [20] was guided by Strategies to Enhance Practice (STEP) framework [21], which is a multi-component competency building framework that was developed using principles of community-based participatory research [22], adaptive interventions [23], and systems frameworks [24] and is primarily driven by practical application of the strategies by practitioners in the framework. STEPs is a framework for identifying modifiable SDC components to increase children’s physical activity during SDC time. The STEPs model is conceptualized as a multi-step, adaptive process where foundational programmatic components essential to increasing attendee’s MVPA are identified and modified. Importantly, STEPs allows for local tailoring to individual SDC needs, recognizing that no two SDCs are alike and different strategies or resources may be necessary for different SDCs to increase children’s MVPA. The model is also designed so that SDCs can enter into the process anywhere along a continuum of support. This allows for SDCs with varying levels of physical activity promotion to benefit from implementation of STEPs.

Strategies to increase children’s physical activity while in attendance at SDCs were guided by the Theory of Expanded, Extended, and Enhanced Opportunities [25]. This theory posits that the primary mechanism for increasing children’s accumulation of physical activity is through the provision of opportunities to be physically active. The Theory of Expanded, Extended, and Enhanced Opportunities includes three broad mechanisms for target by an intervention to increase children’s MVPA. These include: 1) expanding physical activity opportunities (i.e., adding new physical activity opportunities), 2) extending physical activity opportunities (i.e., allocating additional time for existing physical activity opportunities), and 3) enhancing physical activity opportunities (i.e., augmenting existing physical activity opportunities to maximize the amount of physical activity youth accumulate). The utility of the Theory of Expanded, Extended, and Enhanced Opportunities is in its practical application of identified mechanisms for increasing children’s MVPA in any setting, including summer SDCs.

In the Spring of 2016 (February-May), intervention staff met with all SDC program leaders to develop physical activity schedules. The objective was for programs to provide a minimum of three hours of scheduled physical activity time each day. The rationale for providing three hours was based on baseline data that showed, on average, children spent approximately 1/3 of time scheduled for physical activity engaged in MVPA [26]. Thus three hours are necessary to achieve the 60 min/d guideline. The primary intervention strategy to accomplish this goal was to extend existing physical activity opportunities to three hours, given all SDCs had PA opportunities scheduled at baseline (summer 2015). When it was not possible to extend PA opportunities, SDCs were encouraged to expand PA opportunities by integrating PA into enrichment time through short activity breaks (e.g., energizers, brain breaks, etc.). SDCs were also encouraged to expand PA opportunities by exchanging inactive field trips (e.g., movie theatre) for more active field trips (e.g., pool, park).

Finally, all staff employed at intervention SDCs participated in a single 90-minute professional development training covering the topic of enhancing the amount of activity children accumulated during activity opportunities prior to the start of the SDC (May 2016). The professional development training was experiential in nature and founded on the integration of the LET US (i.e., line, elimination, team size, uninvolved staff/children, and space, equipment and rules) Play principles into common games in SDCs [27–31]. These principles help staff identify and modify components of commonly played games that may limit children’s physical activity. During training research staff played common games with SDC staff and identified ways to modify games in order to comply with the LET US Play principles.

In addition, each intervention SDC was visited twice prior to outcome data collection for booster sessions during June 2016. Booster sessions occurred during SDC time, and consisted of a walkthrough of the SDC with the SDC program leader by a trained research staffer. During this walk through, physical activity opportunities were observed for the concepts of expansion (i.e., activity breaks and active field trips), extension (i.e., 3 hours of physical activity opportunities scheduled), and enhancement (i.e., activities played follow the LET US Play principles). Following this 60-minute walk through, the SDC program leader and research staff met for approximately 30 minutes to discuss strategies to address challenges observed with increasing children’s MVPA.

### Measures

#### Outcome evaluation

All measurement occurred during the summer of each project year (July-August). Consistent with previously established protocols [26], children’s physical activity was captured on four nonconsecutive unannounced days (Monday-Thursday). On these days ActiGraph accelerometers (version wGT3X-BT and Link—Shalimar, FL) were placed on children’s non-dominant wrist immediately upon arrival to the camp in the morning. The time each child received an accelerometer was recorded (arrival time) by research staff and children were allowed to participate in regularly scheduled activities throughout the SDC, including all water based (e.g., swimming, splash pad) activities. Prior to departure at the end of the day, research staff removed accelerometers from children and recorded the time (departure time). Cutpoints associated with children’s MVPA (≥530 counts/5 seconds—[32]) and sedentary behaviors (<202 counts/5 seconds—[33]) were applied to distill the data using five second epochs. A valid day of wear time (departure time minus arrival time) was considered greater than 240 minutes, or attendance at the SDC for approximately half the day. SDCs were also visited on an additional two days at the beginning of summer (first two weeks of June) and two days at the end of summer (first two weeks of August) for height and weight measurements. During these visits children’s height (cm) and weight (kg) were measured using standard protocols with children removing their shoes [34]. BMI was calculated and transformed into age and gender specific percentiles [35]. Demographics (i.e., age, race, sex) were also collected at this time. Assent was obtained from all children on each data collection day. Due to the observational nature of the data collected an opt-out protocol was used to obtain passive consent from participating children’s parents. Prior to data collection SDCs provided parents with informational fliers about the data collection procedures, and directions on how to opt out of the study.

#### Process evaluation

Process evaluation data were collected concurrently with outcome data on each data collection day (i.e., the same four non-consecutive unannounced days). Implementation of the Theory of Expanded, Extended, and Enhanced Opportunities components were measured via the System for Observing Staff Promotion of Activity and Nutrition (SOSPAN) [36]. Trained observers, which were blinded to all SDCs treatment condition, rotated through pre-defined target areas while completing continuous scans from the beginning (morning, ~7:30AM) until the end (afternoon, ~6:00PM) of the SDC. An average of 1,182 scans were collected at each SDC each summer across the 4 observation days. Training on the SOSPAN instrument included classroom sessions, video analysis, and field practice prior to data collection. Classroom sessions included orientation to the instrument, a review of study protocols, and committing observational codes to memory. Video analysis included the review and rating of video clips from SDCs using established protocols. Field practice/reliability scans were completed on at least four days in SDCs that were not participating in the study (i.e. three hours per day) prior to the start of data collection. Inter-rater agreement criteria were set at >80% using interval-by-interval agreement for each category, and reliability was collected prior to measurement and each day of data collection [36, 37]. Inter-observer reliability was estimated via percent agreement and weighted kappa (κw). Percent agreement ranged from 93.9–99.8% (median = 98.3) and κw ranged from 0.50–0.92 (median 0.77). Reliability was checked daily to identify disagreements. Operational definitions of variables with borderline or low reliability (<90% agreement) were discussed with observers to ensure acceptable reliability and prevent observer drift.

#### Expand

Implementation of expanded physical activity opportunities was measured via two variables captured by the SOSPAN instrument. First the occurrence of activity breaks, were coded during each SOSPAN scan. Activity breaks are defined as: brief activities (3–5 minute), led by a staff member that occur during an otherwise sedentary time in a non-active setting (e.g., during enrichment taking place in a classroom). Second, SDCs were encouraged to expand activity time in their field trips by replacing non-active field trips (e.g., movies) with active field trips (e.g., parks/playground, pools). A field trip was defined as any instance when children and counselors left the normal SDC location. Observers accompanied SDCs on field trips and noted the location, duration, and specific details of the activities.

#### Extend

To identify the amount of time SDCs allotted for physical activity and other SDC scheduled activities (e.g., enrichment, meals/snacks) two methods were used. First, a schedule was collected from the SDC each observation day. Second, at the beginning of each SOSPAN scan data collectors indicated in what “context” the SDC was currently engaged. Possible contexts included: meal or snack, enrichment, academics, physical activity, or other (transition, camp assembly, etc.). The percentage of scans completed within a given context was used to identify the percentage of a SDCs’ schedule allocated for each context. Percentage of scans in each context was compared to written SDC schedules to identify any discrepancies between written and observed schedules. When discrepancies occurred the observed time was used as the indicator of allotted program time.

#### Enhance

A total of 12 LET US Play principles were collected via SOSPAN during PA opportunities. On each day of observation, the percentage of scans during physical activity opportunities were computed for the following: children waiting in line for turn, children eliminated, small sided-games, staff actively engaged in activity, staff verbally encouraging activity, staff leading an activity, choice of two or more activities offered, girl’s provided with their own physical activity opportunities, staff giving instructions on how to play games, children waiting for activity to start (i.e., idle time), staff withholding physical activity as punishment, and staff disciplining children with physical activity. The distribution of each of the LET US Play principle was divided into tertiles based on the 33rd and 66th percentile for the principle at baseline. SDCs were assigned a one (<33rd centile), two (33rd to 66th centile), or three (>66th centile) for each principle. Scores were summed to represent an overall LET US Play implementation score at baseline and outcome (possible range of scores = 12–36 points).

### Statistical analysis

All analyses were conducted using STATA (v. 14.1, College Station TX). Children with at least one valid day of accelerometer wear at baseline or outcome were included [38–40]. All models employed full-information maximum likelihood estimators to account for missing outcome data. Prior to primary analyses descriptive statistics (i.e., means, standard deviations, percentages—dichotomous variables) were computed for boys’ and girls’ demographics, physical activity levels, and sedentary behaviors separately. Multi-level mixed effects logistic regression models with a treatment group (i.e., difference between groups at baseline), time (i.e., change in physical activity levels in both groups over time), and group-by-time interaction (i.e., differential change between groups over time) main effects were used to estimate the impact of the intervention on the percentage of girls and boys meeting the 60 min/d guideline, separately. The group-by-time interaction was interpreted as the main intervention effect with a positive coefficient indicating a differential change in favor of the intervention for MVPA. These models accounted for the nested nature of the data with days nested within children nested within SDC. The dependent variable was dichotomized with “1” representing children that achieved the 60 min/d of MVPA guideline and “0” representing children that did not achieve the guideline. Age, race, and SDC enrollment/size were included in the model as covariates. Secondary analyses estimated the minutes of MVPA and sedentary time using multi-level mixed effects linear regression models with the same nesting structure and covariates as the primary analyses. All analyses were intent-to-treat.

## Results

### Outcome evaluation

**Fig 1:**
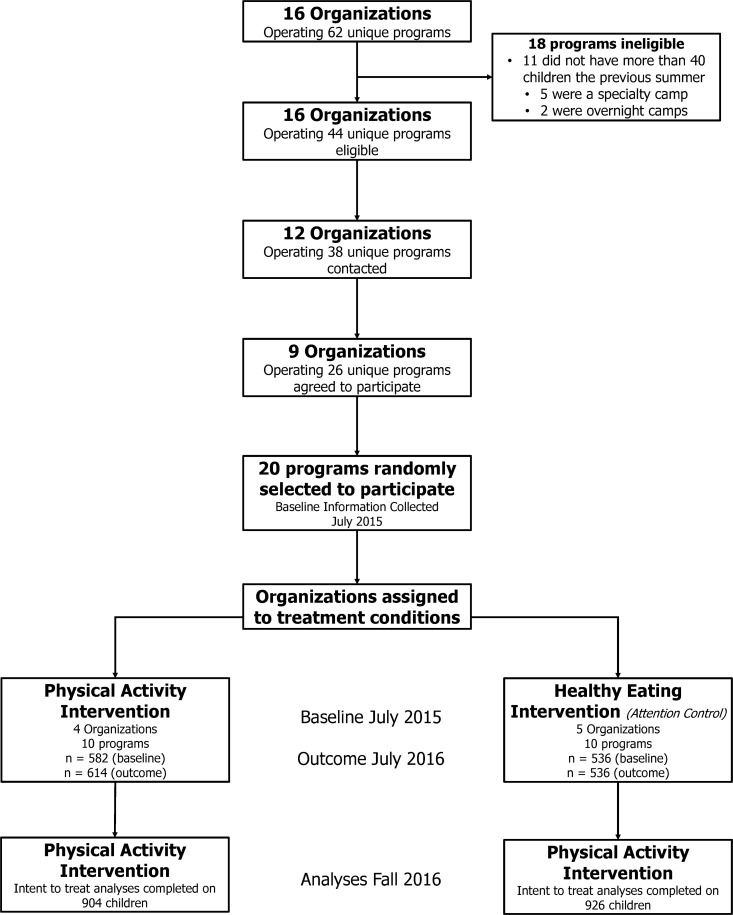
Flow chart of participants for recruitment, data collection, and analyses.

Characteristics of the SDCs and children at baseline and outcome, along with the number of children enrolled and meeting the wear-time inclusion criteria, are presented in Table 1. & Fig 1 details the flow of SDCs and children from recruitment to final outcome evaluation. Change in the percent of children meeting the 60 min/d guideline (primary outcome) and min/d in MVPA and sedentary are presented in Table 2.

**Table 2:** Model implied and raw changes in physical activity levels of children during Summer Day Camp (SDC) program time.

Boys ^a^		2015	2016	Δ	Group-by-time interaction	95% CI		Groupp-value ^b^	Time p-value ^c^	Group-by-time interaction p-value ^d^	Group-by-time odds ratio	95% CI		p-value group-by-time odds ratio
Percentage Meeting the 60 min/day of Moderate-to-Vigorous Physical Activity Standard	Control	81.0%	82.6%	1.6%										
	Intervention	78.0%	88.7%	10.6%	9.0%	(2.6%,	15.5%)	0.543	0.483	0.006	2.04	(1.10,	3.78)	0.023
Moderate-to-Vigorous Physical Activity min/day	Control	98.7	103.0	4.3										
	Intervention	90.4	102.1	11.8	7.5	(0.5,	14.4)	0.22	0.082	0.036				
Sedentary min/day	Control	259.0	274.3	15.3										
	Intervention	266.9	281.3	14.4	-1.0	(-12.1,	10.2)	0.469	≤0.001	0.867				
Girls ^a^														
Percentage Meeting the 60 min/day of Moderate-to-Vigorous Physical Activity Standard	Control	76.1%	70.6%	-5.5%										
	Intervention	69.4%	82.0%	12.6%	18.1%	(9.8%,	26.3%)	0.215	0.08	≤0.001	3.84	(2.02,	7.33)	≤0.001
Moderate-to-Vigorous Physical Activity min/day	Control	85.1	77.6	-7.5										
	Intervention	77.4	90.2	12.8	20.2	(14.1,	26.4)	0.142	≤0.001	≤0.001				
Sedentary min/day	Control	275.1	295.5	20.4										
	Intervention	273.0	280.2	7.2	-13.2	(-25.2,	-1.2)	0.854	≤0.001	0.032				

^a^ Estimates adjusted for age (years), camp enrollment, and race (White non-Hispanics vs. Other).

^b^ Statistically significant value would indicate intervention and control have different values at baseline.

^c^ Statistically significant value would indicate that values for intervention and control are changing over time.

^d^ Statistically significant value would indicate that intervention and control are changing differently over time (interpreted for intervention effect).

At outcome boys and girls attending the intervention camps were 2.04 (95CI 1.10, 3.78) and 3.84 (95CI 2.02, 7.33) times more likely to meet the 60 min/day guideline than boys and girls attending attention control camps, respectively. There were no significant main effects for group. For time there were significant main effects for boys and girls sedentary min/d, and girls MVPA min/d. These indicate that both boys and girls in intervention and control increased sedentary min/d from baseline to outcome and girls in both intervention and control increased MVPA min/d from baseline to outcome. For the group-by-time interaction there were significant main effects for the proportion of boys and girls meeting the MVPA guideline, the and the MVPA min/d. These differences favored the intervention. For girls there was also a significant main effect for sedentary min/d favoring girls at intervention SDCs. Thus, at outcome boys and girls attending the intervention SDCs were 2.04 (p-value = 0.02) and 3.84 (p-value≤0.001) times more likely to meet the 60 min/d guideline than boys and girls attending attention comparison SDCs, respectively. This increase in the likelihood of meeting the guideline was driven by a 10.6% and 12.6% increase in the percent of boys and girls meeting the 60 min/d guideline, respectively, in intervention SDCs. Over the same time period a small increase in the percent of boys (+1.6%) and a decrease in the percent of girls (-5.5%) meeting the 60 min/d guideline was observed in attention control SDCs. Increases of 11.8 and 12.8 min/d of MVPA were observed for boys and girls attending the intervention SDCs, while an increase of 4.3 min/d of MVPA was observed for boys and a decrease of 7.5 min/d of MVPA was observed for girls in the attention control SDCs. Boys and girls in both intervention and attention control SDCs increased time sedentary from baseline to outcome. However, girls in intervention SDCs increased by 13.2 minutes less (7.2 vs. 20.4) than those in attention control SDCs. The time boys’ spent sedentary increased by 14.4 and 15.3 minutes from baseline to outcome in intervention and control SDCs, respectively. Change in the minutes of MVPA accumulated for each SDC is presented separately for boys and girls in **Table 3**. For boys, a total of 8 of the 10 intervention SDCs increased min/d of MVPA compared to 5 of the 10 attention control SDCs. Similarly, for girls, a total of 9 of the 10 intervention SDCs increased min/d of MVPA compared to 4 of the 10 attention control SDCs

**Table 3:** Individual SDC changes.

	Girls			Boys		
Control programs	2015	2016	Δ	2015	2016	Δ
SDC 1 –Association 1	83.9	75.6	-8.3	80.5	82.7	2.2
SDC 2 –Association 1	76.7	57.9	-18.8	94.6	77.6	-17.0
SDC 3 –Association 1	73.4	70.4	-3.0	81.6	78.6	-3.0
SDC 4 –Association 2	97.0	107.3	10.3	122.5	129.2	6.7
SDC 5 –Association 2	70.3	98.3	28.0	71.7	113.4	41.7
SDC 6 –Association 2	112.1	78.6	-33.5	134.0	98.4	-35.6
SDC 7 –Association 2	90.7	81.6	-9.1	115.3	116.4	1.1
SDC 8 –Association 2	72.8	84.0	11.2	97.3	159.0	61.7
SDC 9 –Association 3	75.3	66.9	-8.4	89.8	80.9	-8.8
SDC 10 –Association 4	111.9	77.6	-34.2	114.1	94.3	-19.8
Intervention programs						
SDC 11 –Association 5	86.7	92.2	5.5	104.8	109.7	4.9
SDC 12 –Association 5	74.5	96.9	22.5	77.7	101.9	24.2
SDC 13 –Association 5	82.8	79.8	-3.0	108.1	104.7	-3.4
SDC 14 –Association 5	61.9	85.5	23.6	72.6	101.9	29.2
SDC 15 –Association 6	53.6	71.1	17.6	57.2	78.8	21.6
SDC 16 –Association 6	65.7	93.6	27.9	79.9	101.7	21.8
SDC 17 –Association 7	77.1	88.1	11.0	93.5	100.6	7.1
SDC 18 –Association 8	92.2	93.0	0.8	107.1	104.3	-2.8
SDC 19 –Association 9	90.3	105.3	15.0	93.9	101.8	7.9
SDC 20 –Association 9	85.8	90.8	5.0	97.6	109.1	11.5

All estimates are raw MVPA min/d means

### Process evaluation

Implementation of expanded, extended, and enhanced physical activity opportunities are presented in **Table 4**. Across both intervention and control SDCs, the total daily operating time increased from baseline to outcome by 37 and 38 minutes, respectively. Intervention SDCs increased the percentage of the daily schedule allocated for physical activity (i.e., extension) by 9.9% (~67 minutes), while control SDCs increased by 0.8% (~5 minutes). Observers accompanied SDCs on 38 field trips lasting for an average of two hours and 50 minutes (range = 45 minutes to 6 hours and 30 minutes) across both summers. Intervention SDCs went on eight active field trips at baseline and at outcome, while control SDCs took fewer active fieldtrips at outcome (n = 1) than at baseline (n = 3). The number of days that activity breaks were observed in intervention SDCs increased by 15 (1 at baseline to 16 at outcome) while activity breaks were observed on six more days in control SDCs (3 at baseline to 9 at outcome). Finally, seven of the 10 intervention SDCs increased their average LET US Play implementation score while only three of 10 control SDCs increased from baseline to outcome. This resulted in an average increase for intervention SDCs of +1.2 points (20.4 to 21.6) while control SDCs reduced their implementation score by -2.6 points (22.5 to19.9), from baseline to outcome.

**Table 4:** Implementation of Expanded, Extended, and Enhanced Physical Activity Opportunities.

	Intervention			Control		
	Baseline	Outcome	Δ	Baseline	Outcome	Δ
**Expand**						
Total field trips (n)	12	13	1	9	4	-5
Active field trips (%)	8	8	0	3	1	-2
Total days activity breaks observed	1	16	15	3	9	6
**Extend**						
Daily operating time (min)	635	673	38	617	654	37
Percentage of daily schedule allocated for:						
Meal or Snack (%)	16.5%	14.4%	-2.1%	16.6%	15.2%	-1.4%
Enrichment (%)	46.9%	42.1%	-4.8%	36.2%	42.2%	+6.0%
Academics (%)	2.3%	1.7%	-0.5%	3.6%	1.4%	-2.2%
Other (%)	9.2%	6.7%	-2.5%	9.5%	6.3%	-3.3%
Physical Activity (%)	25.2%	35.1%	+9.9%	34.1%	34.9%	+0.8%
- Free play (%)	13.2%	19.8%	+6.6%	14.8%	14.3%	-0.5%
- Organized (%)	12.0%	15.3%	+3.3%	19.3%	20.6%	+1.3%
**Enhance**						
LET US Play index score	20.4	21.6	1.2	22.5	19.9	-2.6
Number of programs that increased LET US Play index score		7			3	

## Discussion

SDCs have tremendous potential to help the 14 million children[4] they serve annually in the U.S. meet their daily recommended 60 minutes of MVPA. This potential is recognized by several national organizations that have adopted standards calling for SDCs to provide children with all of their daily recommended 60 minutes of MVPA [1–3]. This study is among the first to evaluate the effects of a theory-based, multi-component intervention on the proportion of children meeting the MVPA guideline in the SDC setting. The intervention resulted in a greater likelihood for boys and girls to meet the MVPA guidelines, with eight of 10 intervention SDCs increasing the average minutes of MVPA accumulated by boys and nine of 10 interventions SDCs doing the same for girls. Further, at outcome 88.7% of boys and 82.0% of girls in intervention SDCs were meeting the MVPA guideline, representing a 10.6% and 12.6% increase in the proportion of boys and girls, respectively, meeting the 60 MVPA min/d guideline at outcome. If this increase was extrapolated to all 14 million children attending SDCs it would mean an additional 749,000 and 882,000 boys and girls respectively, would meet this important public health guideline. These findings suggest that SDCs can play a major role in providing all children in attendance with their daily recommended amount of MVPA.

To the authors’ knowledge, this is the first large-scale intervention to target increasing attendee’s accumulation of MVPA at pre-existing SDCs. Previous interventions have targeted specific sub-groups, such as overweight or obese children [12–14] or low income adolescent girls [10] and were created and operated by research personnel [41, 42]. While these interventions have been shown to positively influence health behaviors, their reach and sustainability over time is limited (i.e., once the grant funding ends the camp ends). Targeting pre-existing SDCs expands the potential reach and generalizability of interventions for this setting, since pre-existing SDCs are numerous, serve a diverse population of children, and have extensive contact time with the children attending [4].

This study was guided by the Theory of Expanded, Extended, and Enhanced Opportunities, which focuses on simple and practical strategies to increase children’s physical activity. The three main strategies were to: 1) expand physical activity opportunities (i.e., adding new physical activity opportunities), 2) extend physical activity opportunities (i.e., allocating additional time for existing physical activity opportunities), and 3) enhance physical activity opportunities (i.e., augmenting existing physical activity opportunities to maximize the amount of physical activity youth accumulate). Intervention SDCs successfully extended current physical activity opportunities with an average increase in scheduled time dedicated to physical activity of 9.9% (~67 minutes). Increases in the quality of activities offered were also observed with 7 intervention programs increasing their LET US Play index score from baseline to outcome. Intervention SDCs also successfully added activity breaks to their schedules (1 vs. 16 activity breaks observed from baseline to outcome). The strategies of expanding, extending, and enhancing physical activity opportunities appear to be in the control of staff and program leaders and readily modifiable with proper guidance and support. Further, the large increases in both boys and girls MVPA in intervention SDCs highlight the utility of these strategies to increase physical activity.

It is important to recognize that all SDCs, both intervention and control, provided substantial amounts of MVPA for both boys and girls prior to the intervention (see Table 2). This is likely because SDCs are dedicating significant amounts of program time to physical activity opportunities. At baseline and outcome, control SDCs dedicated almost four hours per day to physical activity opportunities on average, with six programs dedicating three or more hours to physical activity. Intervention SDCs dedicated 2.6 hours to physical activity at baseline with only two programs dedicating more than three hours to physical activity. Correspondingly, on average, boys in intervention and control SDCs accumulated over 1.5 times the daily recommended amounts of MVPA, with girls in both treatment groups accumulating well over the 60-minute recommendation as well. This finding highlights the potential of the SDC setting for helping all children accumulate health enhancing levels of physical activity. Further, with minimal assistance, SDCs may be able to provide all attendees with their daily recommended amounts of MVPA. This is especially important as summer vacation is a “window of vulnerability” and is recognized as a period of extended time (typically 3 months) in which numerous negative effects on a child’s health can occur [5, 8]. Identifying strategies to provide children access to SDCs and then providing support could play an important role in mitigating these negative health impacts.

The current study has several strengths. First, this study is one of the largest and most diverse in terms of SDC operating location (i.e., church, school, community center) and the children served (i.e., 1,830) to evaluate an intervention in the SDC setting targeting children’s physical activity. Further, the use of an objective measure (accelerometers) and including water activities is a unique strength of this study [11]. The rigorous design of the study indicates that changes in MVPA of the children attending the intervention SDCs are likely due to the intervention. However, there are important considerations that must be taken into account when interpreting the findings of this study. First, while there was a control group, SDCs were not randomized to intervention condition. Randomization was intended but not possible due to the constraints of working with multiple camps serviced by the same food provider. Further, while the sample was large and diverse, it was restricted to camps in one southeastern U.S. state.

## Conclusions

The results of this repeated cross-sectional quasi-experimental study suggest that the STEPs approach is promising for assisting SDCs in their attempt to meet physical activity guidelines. SDCs are an important setting for providing children with health enhancing levels of MVPA, and, with additional support, camps have the potential to provide all children attending 60 minutes of MVPA per day.

## Supporting information

S1 File Institutional Review Board Approval Letter. (PDF)

S2 File Study Protocol. (DOCX)

S3 File Clinical Trials Registration Information. (PDF)

S4 File Trend Checklist. (PDF)
