# Supplementary material for: First year physical activity findings from turn up the HEAT (Healthy Eating and Activity Time) in summer day camps
Source: PLoS One. 2017 Mar 28;12(3):e0173791. doi: 10.1371/journal.pone.0173791 (PMC5369693; doi:10.1371/journal.pone.0173791)
Supplement: S2 File — (DOCX) [file pone.0173791.s002.docx]

**Turn Up the HEAT – Healthy Eating and Activity Time in Summer Day Camps**

**Describe the educational setting in which the research will be conducted in:** The research will take place in 20 summer day camp programs run by various organizations across South Carolina. The programs take place during the summer for the entire day (8am-5pm, 10-11wks), are located in various community settings (schools, faith-based, community centers) and provide a combination of scheduled activities which commonly include snack and lunch, enrichment activities (e.g., arts and crafts, music), and opportunities for children to be physically active.

Children attending the programs range in age from 5-12yrs. Their attendance is transitory, with a range of attendance levels from 4 day per week for 1 week during the summer to every day of entire 10 to 11 week summer. Because of these attendance patterns, we are requesting a waiver of informed consent (see below for additional details).

The proposed study and data collection protocol is identical with our previously approved IRB applications for our work in this setting. Please see IRB applications Pro00016542, Pro00006940, Pro00007719, Pro00004484.

Consistent with our previously approved IRB applications, all parents will be informed of the study and measurements taking place in the summer day camps. Information will be posted on summer day camp websites, sent home with parents, and posted in high-traffic areas (e.g., child check-out, drop-off and pick-up) at the programs. A copy of this letter is included in the IRB application.

The current project focuses on organizational changes related to promoting physical activity and the nutritional quality of foods served in summer day camps that serve children ages 5-12yrs. The primary emphasis of the **4 year project** (NIH funded) is to evaluate policies that specify the amount of physical activity children accumulate while attending a summer day camp and the nutritional quality of the foods served/consumed. To achieve this, one of the major components will be professional development training provided to summer day camp staff that are responsible for the daily operations of these programs. Hence, our research team does not directly interact with the children attending the summer day camps, but rather works with summer day camp staff and leaders to help them make changes that translate into changes in children’s activity and healthy eating.

One of the primary evaluations of the policies will be changes in children’s body mass index, physical activity, and the types of foods they eat while attending the programs. Within these programs, children attended on a voluntary basis and at any one site visit (i.e., measurement occasion) a substantial number of children in attendance may be different than those that were present in prior measurement occasions. We are requesting a waiver of informed parental consent for this project due to the transient nature of the children who attend these programs. Hence, the ability to obtain written consent (from parents) can greatly impede to ability to conduct the evaluation of the policies.

Because of the intermittent attendance patterns of children, our primary outcome is site level aggregates of BMI, physical activity, and nutrition. Hence, we will not track individual children over time – this will only be done in the instance where children attend the summer day camp on a consistent yearly basis. We fully expect some kids to be routinely attending, while others will only be present at one measurement occasion.

The decision to waive parental consent is based on our prior experiences working with afterschool programs and summer day camps and is consistent with our previously approved IRB applications for work in this setting – please see Pro00016542, Pro00006940, Pro00007719, Pro00004484. Our previous attempts to collect child information to evaluate the afterschool and summer programs were hindered by the inability to adequately determine which children had received parental consent. Consistent with all of our protocols for this setting, we will ask each child to verbally assent to participating in the data collection.

**Educational practice that is the focus of the research study:** We will be evaluating community-based summer day camp programs’ implementation of physical activity and nutrition policies. These policies focus on the amount of physical activity children should accumulate while attending the programs and the nutritional quality of foods served.

**Study design and methods to be used:** The study design will be a randomized controlled trial, with a wait-listed control. This study will consist of baseline and multiple post-intervention assessments. The following methods will be used to evaluate the changes. All methods have been selected in partnership with the summer day camps and have been used in our prior IRB approved studies.

All parents of children enrolled in the summer day camps will be informed of the study by the summer day camp staff, and information regarding the study will be printed in summer day camp catalogs and located in and around the summer day camp sign-up locations. All parents, children, and staff will be informed of the direct observation protocol and the changes taking place at the summer day camps related to healthy eating and physical activity. Parents not wishing their child to take part in the evaluation will provide the name of their child to the summer day camp providers and that name will be placed on a list of children not to include in the evaluation. Upon parental approval, each child will be asked for verbal assent

Prior to all measurement collection, each child will be asked whether they want to participate (verbal assent). Children indicating “yes” will have the following measure taken. Children indicating “no” will participate in their routine programming.

Child Level Measures: Upon arriving at the programs, participants will have the following physical activity measures collected:

1. Child arrives at the summer day camp
2. Measure height and weight (once at the beginning of summer and again at the end of summer – for those children present at each occasion)
3. Place an accelerometer around the waist of each child using an elastic band
4. Child participates in regularly scheduled activity
5. Collect the accelerometer prior to the child leaving
6. Observer and record the foods and beverages children bring to the summer day camp for snacks/lunch and observe their consumption of these foods

The average duration a child is in attendance for one day is ~7/8hrs during the summer day camp. We anticipate measuring their physical activity levels during this entire time. Up to a total of four days of monitoring will be performed at each measurement occasion (once per year over three years – baseline and 2 years of post-assessment). We have enough monitors to accommodate ~ 180 attendees per day.

To maintain confidentiality, height and weight will be measured in a private location at the programs. Male staff will measure boys and female staff will measure females. The total time to complete these assessments is estimated at 10min maximum.

There are minimal, if any, risks involved with participation in the study. The monitors are unobtrusive, affix the waist using an elastic band, and are relatively unnoticeable once placed. The waist band will be placed over clothes (i.e., shirts) to avoid direct contact with the skin.

All measures will be administered by a project staff member, and these persons will be trained to encourage participants not to do anything that makes them uncomfortable. They also will be trained to conduct the anthropometric measures in a very sensitive and discrete manner. Each data collection team will include at least one female staff member, and female staff will collect all anthropometric data from female students.

Although there is a small risk in all studies that involve human subjects that data will be accessed by an unauthorized person, rigorous safeguards will be put in place to ensure the safety and integrity of the data. Each child will be assigned a numeric identifier, and this identifier, not a name, will be associated with each child’s data. Data will be kept in secure computer files and file cabinets, and access will be limited to the principal investigator.

We will also observe and record (using direct observation) other types of snacks children bring from home and eat while at the programs.

Site Level Measures. We will collect information related to the type of foods/beverages served by each summer day camp (site level measure). This will be done through weekly written menus and collection of receipts for purchasing foods/beverages completed by staff as part of their routine job responsibilities. We will also collect key informant interviews (qualitative data) to evaluate leadership support, adoption/implementation of policies, and barriers and facilitators of policy uptake. No identifiable information will be collected from these individuals to link to their responses.

Staff Level Measures. Each participating counselor will be asked to voluntarily complete a questionnaire related to their skills to promote physical activity and healthy eating of children. Questionnaires will be administered once during each year of evaluation. Completion of the questionnaire will be at the discretion of the counselor.

Staff will be asked to complete a short questionnaire related to the quality of the professional development training they receive.
