# Supplementary material for: First year physical activity findings from turn up the HEAT (Healthy Eating and Activity Time) in summer day camps
Source: PLoS One. 2017 Mar 28;12(3):e0173791. doi: 10.1371/journal.pone.0173791 (PMC5369693; doi:10.1371/journal.pone.0173791)
Supplement: S3 File — (PDF) [file pone.0173791.s003.pdf]

ClinicalTrials.gov
A service of the U.S. National Institutes of Health
Now Available: Final Rule for FDAAA 801 and NIH Policy on Clinical Trial Reporting

Turn up the HEAT - Healthy Eating and Activity Time in Summer Day Camps (HEPA)

This study is ongoing, but not recruiting participants.

Sponsor:
University of South Carolina

Information provided by (Responsible Party):
University of South Carolina

ClinicalTrials.gov Identifier:
NCT02161809

First received: June 10, 2014
Last updated: May 10, 2016
Last verified: May 2016
History of Changes

Purpose

The investigators long-term goal is to increase the number of Summer Day Camps (SDC) that meet the National Afterschool Association Healthy Eating and Physical Activity (HEPA) Standards. The objective is to evaluate the effectiveness of a multi-component intervention designed to increase children's PA levels and improve the quality of lunches and snacks children consume in SDCs. The HEPA intervention is designed to target children, parents, staff, program leaders, and the SDC environment and uses a train-the-trainer model with SDC leaders to train their staff to deliver and integrate the intervention into routine practice. In the proposed study, the investigators will evaluate these HEPA strategies, using a 4-year randomized controlled trial with 20 SDCs. An important feature of the study will be the evaluation of maintenance of the intervention after the removal of research support (e.g., training, boosters) during the final year, as well as costs associated with implementation.

The investigators hypothesize that intervention SDCs will achieve significantly greater increases in HEPA, compared to control SDCs. The investigators expect the study to support the cost-effectiveness and maintenance of our strategies for promoting HEPA that will facilitate SDCs meeting newly-established HEPA standards for this setting.

Condition	Intervention
Childhood Obesity	Behavioral: Healthy Eating and Physical Activity

Study Type: Interventional
Study Design: Allocation: Randomized
Intervention Model: Parallel Assignment
Masking: Open Label

Official Title: Turn up the HEAT - Healthy Eating and Activity Time in Summer Day Camps

Resource links provided by NLM:

MedlinePlus related topics: Health Checkup

U.S. FDA Resources

Further study details as provided by University of South Carolina:

Primary Outcome Measures:

- Changes in percentage of children meeting 60min of MVPA [Time Frame: Year 1 - Year 4] [Designated as safety issue: No]

  Changes in percentage of children at each summer day camp that meet the National Afterschool Alliance Standard of 60 minutes of moderate-to-vigorous physical activity daily.

Secondary Outcome Measures:

- Change in percentage of children bringing/consuming healthy foods/beverages [Time Frame: Year 1 - Year 4]
  [Designated as safety issue: No]

  Trained observers will record the foods/beverages brought/consumed at SDC - snacks and lunches

Enrollment:	1000
Study Start Date:	January 2015
Estimated Study Completion Date:	August 2018
Estimated Primary Completion Date:	August 2018 (Final data collection date for primary outcome measure)

Arms	Assigned Interventions
Experimental: Physical Activity Intervention<br>This arm (10 summer day camps) will receive the Physical Activity intervention the first year and both healthy eating and physical activity the second and thrid years.	Behavioral: Healthy Eating and Physical Activity<br>The Healthy Eating and Physical Activity (HEPA) intervention aims to increase the quality of foods and beverages and physical activity opportunities in summer day camps. Through staff-level training, physical activity opportunities are modified to provide children with a substantial amount of meaningful physical activity. Through working with foods service providers, changes to the types of foods and beverages served will be made.<br>Other Name: HEPA
Healthy EAting Intervention<br>This arm (10 summer day camps) will receive the Healthy Eating intervention the first year and both healthy eating and physical activity the second and thrid years.	Behavioral: Healthy Eating and Physical Activity<br>The Healthy Eating and Physical Activity (HEPA) intervention aims to increase the quality of foods and beverages and physical activity opportunities in summer day camps. Through staff-level training, physical activity opportunities are modified to provide children with a substantial amount of meaningful physical activity. Through working with foods service providers, changes to the types of foods and beverages served will be made.<br>Other Name: HEPA

Detailed Description:

The investigators aim to:

Evaluate the impact of the HEPA interventions on: 1.) The proportion of children meeting the PA Standard (i.e., ≥60min MVPA/d) while attending summer day camp; 2.) The proportion of foods (e.g., fruit, vegetable, water) children and staff bring and consume at the summer day camp that meet the HE Standards; and 3/) Changes in children's age-sex specific BMI percentile from the start to end of summer

Evaluate the cost-effectiveness of the HEPA intervention

Evaluate the maintenance of the HEPA intervention in summer day camps

Eligibility

Ages Eligible for Study:	6 Years to 14 Years (Child)
Genders Eligible for Study:	Both
Accepts Healthy Volunteers:	No

Criteria

Inclusion Criteria:

- Summer Day camps will be eligible if:
- They operate for at least 10 weeks during the summer
- They do not have any primary focus such as sports, art, or tutoring (must be a general camp)
- Enrollment is at least 40 campers
- Operation hours are at least 8 hours.

Exclusion Criteria:

- Children will be unable to wear an activity monitor if experience any physical and/or orthopedic impairment that limits the child's ability to participate in regular PA (e.g., wheelchair user)

Contacts and Locations

Choosing to participate in a study is an important personal decision. Talk with your doctor and family members or friends about deciding to join a study. To learn more about this study, you or your doctor may contact the study research staff using the Contacts provided below. For general information, see Learn About Clinical Studies.

Please refer to this study by its ClinicalTrials.gov identifier: NCT02161809

Locations

United States, South Carolina

University of South Carolina
Columbia, South Carolina, United States, 29201

Sponsors and Collaborators

University of South Carolina

Investigators

Principal Investigator: Michael W Beets, PhD University of South Carolina

More Information

Responsible Party:	University of South Carolina
ClinicalTrials.gov Identifier:	NCT02161809 History of Changes
Other Study ID Numbers:	1R01HD079372-01A1
Study First Received:	June 10, 2014
Last Updated:	May 10, 2016
Health Authority:	United States: Institutional Review Board

Keywords provided by University of South Carolina:
MVPA
Healthy Eating
children
childhood obesity

Additional relevant MeSH terms:
Pediatric Obesity
Obesity
Overnutrition
Nutrition Disorders
Overweight
Body Weight
Signs and Symptoms

ClinicalTrials.gov processed this record on November 21, 2016
