# Supplementary material for: First year physical activity findings from turn up the HEAT (Healthy Eating and Activity Time) in summer day camps
Source: PLoS One. 2017 Mar 28;12(3):e0173791. doi: 10.1371/journal.pone.0173791 (PMC5369693; doi:10.1371/journal.pone.0173791)
Supplement: S1 File — (PDF) [file pone.0173791.s001.pdf]

UNIVERSITY OF
SOUTH CAROLINA

OFFICE OF RESEARCH COMPLIANCE

February 25, 2014

Michael Beets, PhD
Arnold School of Public Health
Department of Exercise Science
921 Assembly, Rm 131
Columbia, SC 29208

Re: **Pro00032881**
Study Title: *Turn Up the HEAT - Healthy Eating and Activity Time in Summer Day Camps*

Dear Dr. Beets:

The referenced study was reviewed and approved by the University of South Carolina Institutional Review Board (IRB) by Expedited review on 2/24/2014 (category **4, 5, & 7**).

Approval is for a one-year period from **2/24/2014 to 2/23/2015**. Approved consent document(s), if applicable, are located under the "Stamped ICF" tab on the Study Workspace screen in eIRB.

University of South Carolina Assurance number: FWA 00000404 / IRB Registration number: 00000204

PLEASE NOTE THE FOLLOWING APPROVAL CONDITIONS

- The research must be conducted according to the proposal/protocol that was approved by the IRB.
- Changes to the procedures, recruitment materials, or consent document must be approved by the IRB prior to implementation.
- *If applicable*, each subject should receive a copy of the approved, date stamped, consent document.
- It is the responsibility of the principal investigator to report promptly to the IRB:
  - Unanticipated problems and/or unexpected risks to subjects
  - Adverse events effecting the rights or welfare of any human subject participating in the project
- Research records, including signed consent documents, must be retained for at least three years after the termination of the last IRB approval.
- No subjects may be involved in any study procedure prior to the IRB approval date, or after the expiration date. For continuing research, an update of the study is required prior to the expiration date. The PI is responsible for initiating the Continuing Review process. At the time a study is terminated (closed) a final report should be submitted to the IRB.

The Office of Research Compliance is an administrative office that supports the USC Institutional Review Board. If you have questions, please contact Arlene McWhorter at arlenem@sc.edu or (803) 777-7095.

Sincerely,

Lisa M. Johnson
IRB Manager

cc: Larry Durstine
